# High density lipoprotein-cholesterol is inversely associated with blood eosinophil counts among asthmatic adults in the USA: NHANES 2011-2018

**DOI:** 10.3389/fimmu.2023.1166406

**Published:** 2023-04-24

**Authors:** Jun Wen, Rongjuan Zhuang, Chunyan He, Mohan Giri, Shuliang Guo

**Affiliations:** Department of Respiratory and Critical Care Medicine, The First Affiliated Hospital of Chongqing Medical University, Chongqing Medical University, Chongqing, China

**Keywords:** serum lipid, high density lipoprotein-cholesterol (HDL-C), eosinophil, asthma, National Health and Nutrition Examination Survey (NHANES), machine learning

## Abstract

**Background:**

A growing number of research strongly suggest that metabolic syndrome and dyslipidemia contribute to the establishment of a pro-inflammatory state in asthma, according to accumulating data. However, the majority of recent research has focused on the association between lipids and asthma in children and adolescents, with contradictory findings. Consequently, we analyzed the relationship between serum lipid and blood eosinophil counts using data from the NHANES in the USA.

**Methods:**

After screening the individuals from the 2011 to 2018 NHANES survey, a total of 2,544 out of 39156 participants were eligible for our study. The potential association was discussed using the linear regression model, XGBoost algorithm model, generalized additive model, and two-piecewise linear regression model. In addition, we ran stratified analysis to identify specific demographics.

**Results:**

After adjusting for covariates, the result indicated that blood eosinophil counts decreased by 45.68 (-68.56, -22.79)/uL for each additional unit of HDL-C (mmol/L). But serum LDL-C, total cholesterol or triglyceride was not correlated with blood eosinophil counts. Furthermore, we used machine learning of the XGBoost model to determine LDL-C, age, BMI, triglyceride, and HDL-C were the five most critical variables in the blood eosinophil counts. The generalized additive model and two-piecewise linear regression model were used to further identify linear relationship between the serum HDL-C and blood eosinophil counts.

**Conclusions:**

Our study elucidated a negative and linear correlation between serum HDL-C and blood eosinophil counts among American asthmatic adults, suggesting that serum HDL-C levels might be associated with the immunological condition of asthmatic adults. There was no correlation between serum LDL-C, total cholesterol, or triglyceride levels and blood eosinophil counts.

## 1 Introduction

Asthma is a chronic respiratory disease with global prevalence, affecting 1–18% of the populations of different countries (1). Asthma is a complex and heterogeneous disorder resulting from multiple inflammatory mechanisms (2). Eosinophilic airway inflammation has emerged as a defining characteristic of one severe form of asthma, to the point where asthma is routinely classified as eosinophilic or noneosinophilic (2). In the activation of eosinophilic and non-eosinophilic-mediated inflammation pathways, eosinophils are the most important cell type (3). More than 80% of patients in a global real-life severe asthma cohort were likely to have an eosinophilic phenotype (4). Eosinophil (Eos) is the predominant inflammatory effector cell in eosinophilic asthma (EA). EA is characterized by elevated eosinophils in peripheral blood and airway tissue (5, 6). In addition, eosinophil counts in the blood correlate with disease severity in EA (7).

Lipid metabolism issues are known to be linked to inflammation. In recent years, a growing number of studies have strongly suggested that metabolic syndrome and dyslipidemia contribute to the development of an asthmatic pro-inflammatory state (8–10). However, the majority of recent research has focused on the association between lipids and asthma in children and adolescents, with contradictory findings (11). The relationship between lipid profiles and asthma in adults has received less attention, and large-scale studies are lacking.

Consequently, the purpose of this study was to examine the relationship between lipid profiles and peripheral blood eosinophil counts in adults with asthma. The National Health and Nutrition Examination Survey (NHANES) was investigated for secondary data analysis. After controlling for a large number of confounding variables, we attempted to clarify the relationship between serum lipid and blood eosinophil counts among asthmatic patients in the United States and to explain the contradictory findings.

## 2 Materials and methods

### 2.1 Data source

Every two years, the National Health and Nutrition Examination Survey (NHANES), conducted by the Centers for Disease Control and Prevention (CDC), collected data on the health and nutritional status of the U.S. population. NHANES utilized a complex, stratified sampling design to select samples of non-institutionalized civilians that are representative of the population. The NCHS Institutional Review Board approved the NHANES database in accordance with the revised Helsinki Declaration. Before the data collection procedures and exhaustive health examinations, the informed consent forms were completed.

### 2.2 Study population

**Figure 1:**
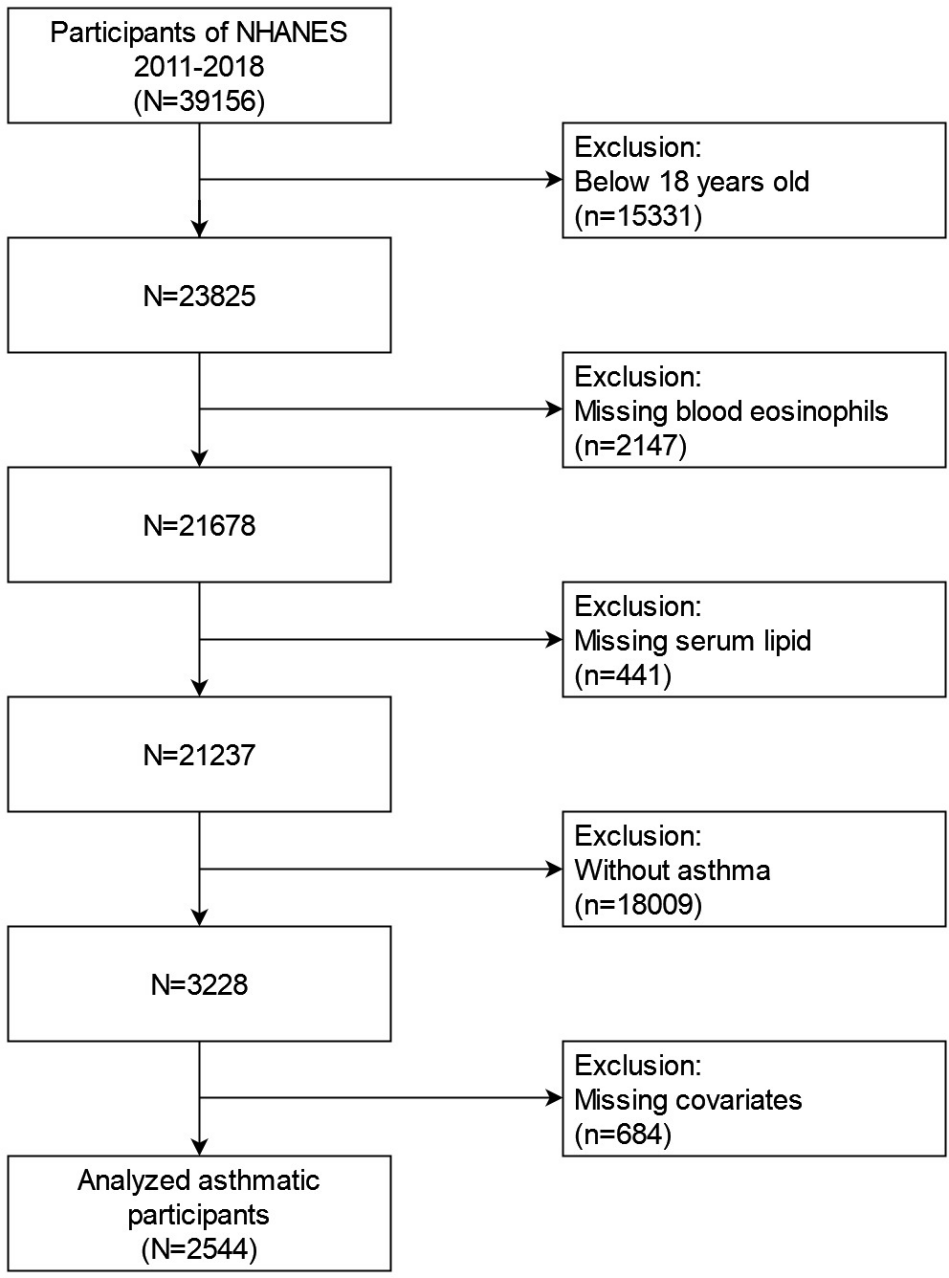
Flowchart for choosing participants with asthma for analysis.

Our study applied data from four NHANES cycles (2011–2018). For the second analysis, these data included demographic data, examination data, laboratory data, diet data, and questionnaire data. When we initially screened the NHANES database, all participants (n=39156) from 2011 to 2018 were included. We included following individuals: (1) age≥18 years old (N=23825) (2) complete blood eosinophil counts data (N=21678) (3) complete serum lipid data (N=21237). Meanwhile, in the process of further screening, the following individuals were excluded: (1) individuals without asthma (n=18009) (2) missing data about covariates at least one of the following (n=684): education level; marital status; poverty to income ratio (PIR); BMI; smoked status; alcohol intake; hypertension history; diabetes history; COPD history. In the end, a total of 2544 asthmatic American adults were attended in our study. **Figure 1** depicts the flowchart for the screening procedure.

### 2.3 Measurement of serum lipid

The lipid profile was evaluated using samples from subjects who had fasted for at least 8.5 h but less than 24 h. Using enzymatic assays, total cholesterol (TC), triglyceride (TG), high density lipoprotein-cholesterol (HDL-C), and low density lipoprotein-cholesterol (LDL-C) levels were determined. On the NHANES website, you can find thorough instructions for specimen collection and processing.

### 2.4 Measurement of blood eosinophil counts

Blood differential counts were performed by using the Beckman Coulter HMX (Beckman Coulter, Fullerton, Calif), a quantitative and automated hematologic analyzer and leukocyte differential cell counter for *in vitro* diagnostic use in clinical laboratories. On the NHANES website, you can find a detailed description of laboratory procedures.

### 2.5 Covariates and asthma assessment

We chose the following variables as potential covariates in our study due to some confounding factors that may affect the results. Participants’ continuous variables consisted of age (years old), poverty to income ratio (grouped by trisection: low, middle, high), BMI (kg/m2) and alcohol intake (gm). And categorical variables were comprised of race/ethnicity (Mexican American, other Hispanic, non-Hispanic white, non-Hispanic black, others), educational level (less than high school, high school, more than high school), marital status (married, single, living with a partner), smoking status (whether smoked at least 100 cigarettes in life), lipid-lowering drugs use (whether lipid-lowering drugs use in past 30 days), antiallergic drugs use (whether antiallergic drugs use in past 30 days; antiallergic drugs include antihistamines and glucocorticoids), hypertension history (Yes, No) and diabetes history (Yes, No, Borderline), and COPD history (Yes, No). The assessment of asthma was based on the information from the questionnaire section of the US National Health Interview Survey. In order to assess asthma, participants were asked, “Has a doctor or other health professional ever told you that you have asthma?” If the participant responds “yes,” he or she was regarded as asthma patient. The official NHANES website (http://www.cdc.gov/nchs/nhanes/) contains a more comprehensive description of the variables.

### 2.6 Statistical analysis

We used complex sample analyses to explore the relationships between serum lipid and blood eosinophil counts among asthmatic adults in the United States, in accordance with CDC guidelines. Continuous and categorical variables were presented separately as means ± SD and percentages. First, eosinophil counts in the blood were transformed into four quartiles. We calculated the p-value of categorical variables using the weighted chi-square test, and the p-value of continuous variables using the Kruskal Wallis rank sum test. If the count variable has a theoretical value less than 10, we calculated the p-value using Fisher’s exact probability test. Second, three weighted univariable and multivariable linear regression models were developed to investigate the associations between serum lipid and blood eosinophil counts. Model I made no adjustments, Model II made adjustments for age, race, and gender, and Model III made adjustments for all variables with the exception of the analyzed variable. In addition, we employed the trend test and the generalized additive model (GAM) to confirm the validity of the regression analysis results. Next, we performed stratified analyses to determine the association between HDL-C and blood eosinophil counts in specific subgroups. To determine whether individual characteristics influenced the association between HDL-C and blood eosinophil counts, interaction tests were conducted. Eventually, the XGBoost algorithm model’s machine learning was intended to determine the relative importance of various variables on the effect of blood eosinophil counts. R software (Version 4.2.0) and the R package were used to conduct all statistical analyses. A p-value 0.05 was considered statistically significant in our study.

## 3 Results

### 3.1 Basic characteristics of the analyzed population

**Table 1** presents the weighted basic characteristics of individuals based on blood eosinophil counts quartiles (Q1-Q4) for a total of 2544 asthmatic adults aged 20 to 80 years old who participated in our study (2011–2018 NHANES). In our investigation, the mean age of the investigated population was 47.3 years old, and the majority of the population was non-Hispanic White. We observed that the distribution of age, gender, race, BMI, lipid-lowering drugs use, hypertension history, HDL-C and triglyceride was statistically significant (p value < 0.05) across all quartiles of blood eosinophil counts. Nevertheless, no significant differences (p value > 0.05) were observed across different quartiles of blood eosinophil counts for education level, marital status, poverty to income ratio, smoking status, alcohol intake, antiallergic drugs use, diabetes history, COPD history, LDL-C and total cholesterol.

**Table 1:** By quartiles of blood eosinophil counts, weighted characteristics of study individuals are disaggregated.

	Q1(0)	Q2(100)	Q3(200)	Q4(300-2200)	P value
Gender (%)					0.0036
Male	33.96	34.36	40.18	45.62	
Female	66.04	65.64	59.82	54.38	
Age,mean ± SD (years)	45.93 ± 1.82	43.43 ± 0.75	46.31 ± 0.8	47.24 ± 0.72	0.0011
Race/ethnicity (%)					0.0271
Mexican American	4.96	4.7	5.79	6.81	
Other Hispanic	5.58	5.69	6.01	6.32	
Non-Hispanic White	63.35	69.27	69.01	68.28	
Non-Hispanic Black	23.44	12.56	10.4	10.85	
Other Race	2.67	7.79	8.79	7.74	
Education (%)					0.2189
Less than high school	14.65	10.27	13.3	12.51	
High school	17.18	19.24	20.18	23.79	
More than high school	68.17	70.49	66.52	63.71	
Marital status (%)					0.321
Married	41.57	52.43	48.48	52.94	
Single	54	39.91	42.98	39.7	
Living with a partner	4.43	7.65	8.54	7.35	
Poverty to income ratio,mean ± SD	2.51 ± 0.23	2.91 ± 0.09	2.74 ± 0.11	2.79 ± 0.09	0.1223
BMI,mean ± SD (kg/m2)	29.32 ± 1.01	29.53 ± 0.33	30.86 ± 0.49	31.7 ± 0.42	0.0002
Smoked at least 100 cigarettes in life (%)					0.0654
Yes	51.1	43.61	45.32	52.52	
No	48.9	56.39	54.68	47.48	
Alcohol,mean ± SD (gm)	14.62 ± 3.62	12.13 ± 1.39	12.23 ± 1.96	12.63 ± 1.67	0.8987
Lipid-lowering drugs (%)					0.003
Yes	13.95	10.36	18.37	14.36	
No	86.05	89.64	81.63	85.64	
Antiallergic drugs (%)					0.1555
Yes	20.74	14.23	16.12	19.07	
No	79.26	85.77	83.88	80.93	
Hypertension (%)					0.0011
Yes	38.55	30.11	37.97	41.88	
No	61.45	69.89	62.03	58.12	
Diabetes (%)					0.0542
Yes	15.91	8.1	13.33	14.72	
No	80.94	88.94	84.36	82.65	
Borderline	3.15	2.96	2.31	2.63	
COPD (%)					0.1067
Yes	15.97	7.68	11.7	9.93	
No	84.03	92.32	88.3	90.07	
Serum HDL-C, mean ± SD (mmol/L)	1.68 ± 0.12	1.45 ± 0.02	1.35 ± 0.02	1.32 ± 0.02	<0.0001
Serum LDL-C, mean ± SD (mmol/L)	2.99 ± 0.12	3.17 ± 0.04	3.21 ± 0.05	3.28 ± 0.05	0.0786
Serum total cholesterol, mean ± SD (mmol/L)	4.93 ± 0.12	4.92 ± 0.04	4.94 ± 0.07	4.98 ± 0.06	0.8635
Serum triglyceride, mean ± SD (mmol/L)	1.3 ± 0.09	1.51 ± 0.05	1.89 ± 0.16	1.89 ± 0.08	<0.0001

Continuous and categorical variables were presented as the means ± SD and percentage, respectively. Q1-Q4: Grouped by quartile according to the blood eosinophil counts. Q1: Eosinophils number is 0 cells/uL; Q2: Eosinophils number is 100 cells/uL; Q3: Eosinophils number is 200 cells/uL; Q4: Eosinophils number is 300-2200 cells/uL. Antiallergic drugs: antihistamines and glucocorticoids. The second analysis of our study included HDL-C, LDL-C, triglyceride, total cholesterol, blood eosinophil counts, demographic data, examination data, laboratory data, diet data, and questionnaire data.

### 3.2 The relationships between serum lipid and blood eosinophil counts

Three weighted univariable and multivariable regression models were used to assess whether the relationships between serum lipid and blood eosinophil counts among American asthmatic adults were statistically significant (**Table 2**). Based on the three models, we observed that only HDL-C was inversely correlated with blood eosinophil counts, with statistical significances. Nevertheless, no significant differences (p value > 0.05) were observed in relationships between LDL-C, total cholesterol or triglyceride with blood eosinophil counts. In the Model I, which didn’t adjust for any variables, the blood eosinophil counts decreased by 49.07 (-65.90, -32.23)/uL for each additional unit of HDL-C (mmol/L). In the Model II, which adjusted for age, gender and race, the blood eosinophil counts decreased by 45.38 (-63.18, -27.58)/uL for each additional unit of HDL-C (mmol/L). In the Model III, which adjusted for age, gender, race, education level, marital status, poverty income ratio, BMI, smoked status, alcohol intake, lipid-lowering drugs use, antiallergic drugs use, hypertension history, diabetes history, COPD history, LDL-C, total cholesterol and triglyceride, blood eosinophil counts decreased by 45.68 (-68.56, -22.79)/uL for each additional unit of HDL-C (mmol/L). Also, we observed the trend test was statistically significant in the Model I and II, whereas not in the Model III (p for trend <0.05), which indicated HDL-C was linear associated with blood eosinophil counts in the Model I and II, but not in in the Model III (**Table 3**). Therefore, we further employed the generalized additive model (GAM) and threshold effect model to assess the nonlinear or linear relationship between HDL-C and blood eosinophil counts in the Model III. Likewise, we also observed that serum HDL-C was also linearly and inversely linked with blood eosinophil counts among adults without asthma (p for trend <0.05), who met inclusion and exclusion criteria (**Supplementary Figure 1**, **Supplementary Table 1**).

**Table 2:** The correlation between serum lipid and blood eosinophil counts in three weighted regression models.

	Non-Adjusted Model	Minimally Adjusted Model	Fully Adjusted Model
	β (95% CI) P value	β (95% CI) P value	β (95% CI) P value
Serum HDL	-49.07 (-65.90, -32.23) <0.0001	-45.38 (-63.18, -27.58) <0.0001	-45.68 (-68.56, -22.79) 0.0004
Serum LDL-C	4.60 (-6.40, 15.59) 0.4157	3.08 (-7.80, 13.95) 0.5816	1.60 (-9.74, 12.94) 0.7836
Serum total cholesterol	-1.50 (-10.34, 7.35) 0.7414	-2.71 (-11.66, 6.24) 0.5554	-5.96 (-24.87, 12.94) 0.5402
Serum triglyceride	4.95 (-0.68, 10.59) 0.0902	3.14 (-1.81, 8.09) 0.2189	-1.51 (-6.36, 3.33) 0.5443

Non-Adjusted Model adjusted for none. Minimally Adjusted Model adjusted for age, race and gender. Fully Adjusted Model adjusted for all variables except for the analyzed variable.

**Table 3:** Weighted regression models and trend tests elucidate the correlation between HDL-C and blood eosinophil counts.

	Model I	Model II	Model III
	β (95% CI) P value	β (95% CI) P value	β (95% CI) P value
HDL-C	-49.07 (-65.90, -32.23) <0.0001	-45.38 (-63.18, -27.58) <0.0001	-45.68 (-68.56, -22.79) 0.0004
HDL-C quartiles			
Q1	Reference	Reference	Reference
Q2	-11.99 (-40.76, 16.78) 0.4173	-8.74 (-36.42, 18.93) 0.5385	2.63 (-29.37, 34.62) 0.8732
Q3	-27.12 (-53.55, -0.69) 0.0489	-21.81 (-48.23, 4.60) 0.1116	0.08 (-31.00, 31.16) 0.9962
Q4	-59.75 (-83.47, -36.04) <0.0001	-52.81 (-76.71, -28.90) 0.0001	-12.07 (-54.92, 30.78) 0.5846
P for trend	<0.0001	<0.0001	0.6254

Model I adjusted for none. Model II adjusted for age, race and gender. Model III adjusted for age, race, gender, education level, marital status, poverty to income ratio, BMI, smoked status, alcohol intake, lipid-lowering drugs use, antiallergic drugs use, hypertension history, diabetes history, COPD history, triglyceride, LDL-C and total cholesterol.

### 3.3 Stratified associations between HDL-C and blood eosinophil counts

To verify the stability of multivariable regression analyses results, we further analyzed stratified associations between the serum HDL-C levels and blood eosinophil counts in a specific subgroup by gender, age, race, education level, marital status, poverty to income ratio, BMI, COPD history, lipid-lowering drugs use and antiallergic drugs use (**Table 4**). According to stratified analysis results, we found an inversely association between HDL-C and blood eosinophil counts in the specific populations with asthma, who were female, age ≥40, Non-Hispanic White people, less or more than high school, single or married status, low or high group of poverty to income ratio, BMI<25 or ≥28, and without lipid-lowering drugs use. Besides, we observed no interactions among all subgroup analyses results (all P values for interaction > 0.05). In addition, we also conducted the stratified analyses of associations between the serum HDL-C levels and blood eosinophil counts in adults with Asthma-COPD Overlap Syndrome (ACOS). We found this inverse association in the specific populations with ACOS, who were female, age ≥60, Non-Hispanic White people, more than high school, single status, low group of poverty to income ratio, and without lipid-lowering drugs use (**Supplementary Table 2**).

**Table 4:** Stratified correlation of serum HDL-C on blood eosinophil counts in the predetermined and exploratory subgroups.

	N	β (95% CI) P value	P-interaction
Gender			0.2577
Male	1044	-27.86 (-70.56, 14.84) 0.2093	
Female	1500	-52.08 (-74.55, -29.61) 0.0001	
**Age**			0.4387
<40	990	-28.71 (-68.16, 10.74) 0.1628	
40-60	796	-51.97 (-78.26, -25.68) 0.0005	
>=60	758	-53.66 (-84.24, -23.09) 0.0016	
**Race**			0.09
Mexican American	232	-19.20 (-88.19, 49.78) 0.5891	
Other Hispanic	264	-6.02 (-81.83, 69.79) 0.8774	
Non-Hispanic White	1107	-57.79 (-83.48, -32.09) 0.0001	
Non-Hispanic Black	625	-29.13 (-65.55, 7.30) 0.1269	
Other Race	316	22.54 (-36.50, 81.57) 0.4598	
**Education level**			0.2058
Less than high school	458	-71.52 (-115.19, -27.85) 0.0029	
High school	547	-29.63 (-73.70, 14.43) 0.1963	
More than high school	1539	-45.23 (-70.17, -20.28) 0.0011	
**Marital status**			0.3335
Married	1151	-40.69 (-66.69, -14.70) 0.0042	
Single	1183	-59.69 (-90.11, -29.27) 0.0005	
Living with a partner	210	-20.97 (-84.75, 42.81) 0.5237	
**Poverty to income ratio**			0.4271
Low	838	-61.77 (-97.67, -25.87) 0.0019	
Middle	858	-34.09 (-70.63, 2.45) 0.0763	
High	848	-47.19 (-74.54, -19.85) 0.0018	
**BMI**			0.3847
<25	615	-43.45 (-76.77, -10.13) 0.0152	
25-28	465	-31.02 (-71.93, 9.90) 0.1465	
>=28	1464	-62.61 (-96.88, -28.34) 0.0011	
**COPD**			0.5734
Yes	273	-54.30 (-89.60, -19.00) 0.0048	
No	2271	-42.42 (-68.94, -15.90) 0.0035	
**Lipid-lowering drugs**			0.6703
Yes	395	-35.72 (-88.08, 16.65) 0.1899	
No	2149	-47.33 (-70.82, -23.83) 0.0004	
**Antiallergic drugs**			0.9169
Yes	426	-47.43 (-86.96, -7.90) 0.0244	
No	2118	-44.94 (-71.89, -18.00) 0.0024	

Above adjusted for age, race, gender, education level, marital status, PIR, BMI, smoked status, alcohol intake per day, lipid-lowering drugs, antiallergic drugs, hypertension, diabetes, COPD history, triglyceride, LDL-C and total cholesterol. In every instance, subgroup analyses were conducted without adjusting for the stratification variable itself.

### 3.4 Evaluating chosen variables’ relative significance by XGBoost algorithm model

**Figure 2:**
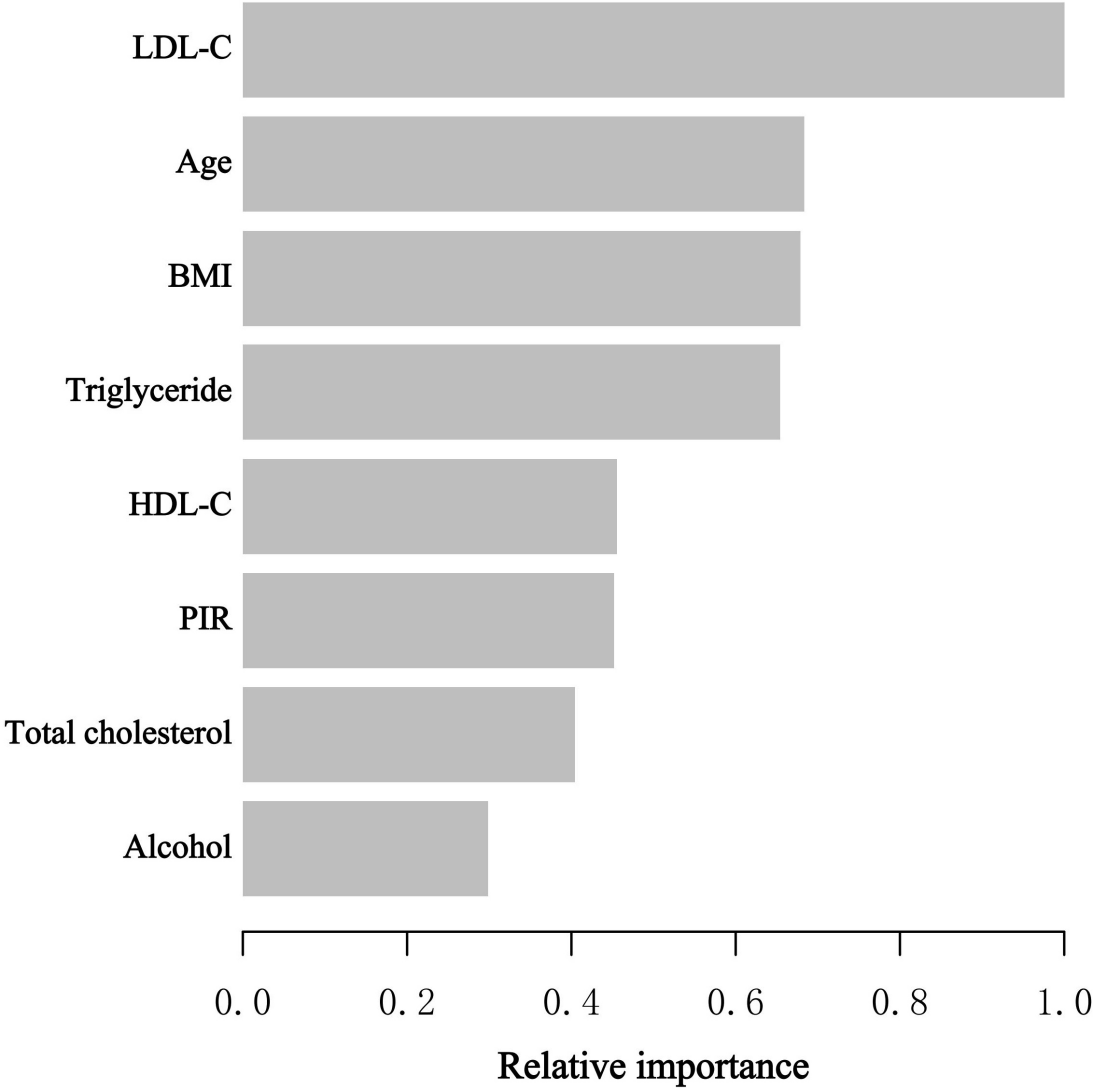
XGBoost algorithm model revealed the relative importance of selected variables on blood eosinophil counts as well as the variable importance score for each variable. The X-axis represented the importance score, the relative number of a variable used to distribute the data, and the Y-axis indicated the selected variables.

During the model development and validation phase, we identified the relative importance of chosen variables correlated with blood eosinophil counts using machine learning. Variables included age, poverty to income ratio (PIR), BMI, HDL-C, LDL-C, total cholesterol and triglyceride. According to the results of XGBoost algorithm model, we observed that LDL-C, age, BMI, triglyceride, and HDL-C were the five most critical variables in the blood eosinophil counts (**Figure 2**). In the end, we selected HDL-C as the relatively critical relative variable to further construct the smooth curve in order to validate the validity of the multivariable regression analyses results.

### 3.5 Verifying the linear relationship between HDL-C and blood eosinophil counts

**Figure 3:**
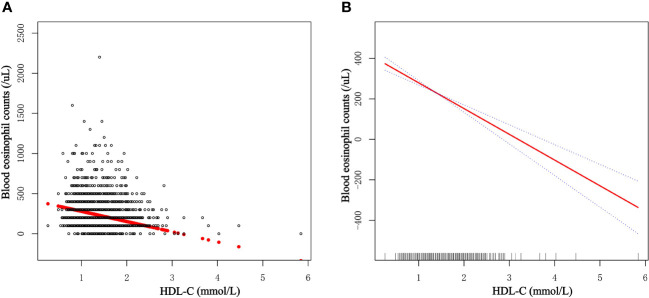
Dose-response relationship of serum HDL-C levels with blood eosinophil counts among asthmatic adults. **(A)** Each dot represents an individual sample. **(B)** The solid red line indicates the smooth fitting curve between serum HDL-C and blood eosinophil counts, whereas the dotted blue line represents 95% confidence intervals of the fitting.

The generalized additive model (GAM) is highly attuned to recognizing linear or nonlinear relationships. To verify the validity of multivariable regression analysis results, we explored the nonlinear or linear relationship between HDL-C levels and blood eosinophil counts using the GAM. Using GAM, we constructed a smooth fit curve to represent the relationship based on Model III (**Figure 3**). We observed the linear and negative relationship between serum HDL-C with blood eosinophil counts in asthmatic adults after adjusting age, race, gender, education level, marital status, poverty to income ratio, BMI, smoked status, alcohol intake, lipid-lowering drugs use, antiallergic drugs use, hypertension history, diabetes history, COPD history, triglyceride, LDL-C and total cholesterol. Similarly, we also observed the linear and negative relationship in adults with ACOS after adjusting age, race, gender, education level, marital status, poverty to income ratio, BMI, smoked status, alcohol intake, lipid-lowering drugs use, antiallergic drugs use, hypertension history, diabetes history, triglyceride, LDL-C and total cholesterol (**Supplementary Figure 2**). What’s more, we utilized the threshold effect model to confirm the linear or nonlinear relationship between HDL-C and blood eosinophil counts in asthmatic adults (**Table 5**). We found that the inflection point was not statistically significant (log-likelihood ratio > 0.05) and that there were no statistically significant differences between the one-line model and the segmented regression model. Therefore, the one-line model was more appropriate to explain the relationship between HDL-C and blood eosinophil counts. All of the aforementioned findings revealed an inverse and linear relationship between HDL-C and blood eosinophil counts among asthmatic adults in the United States.

**Table 5:** Threshold effect analysis of between HDL-C on blood eosinophil counts in asthmatic adults using the two-piecewise linear regression model.

	β (95% CI) P value
Model I	
linear effect	-45.68 (-65.72, -25.63) <0.0001
Model II	
Inflection point(K)	0.85
SUA/SCr< K	116.51 (-107.64, 340.65) 0.3084
SUA/SCr> K	-47.96 (-68.24, -27.67) <0.0001
**Log likelihood ratio**	0.152

The threshold effect analysis model adjusted for age, race, gender, education level, marital status, PIR, BMI, smoked status, alcohol intake, lipid-lowering drugs, antiallergic drugs, hypertension, diabetes, COPD history, triglyceride, LDL-C and total cholesterol.

## 4 Discussion

According to our knowledge, this study is one of the largest cross-sectional studies to explore the relationship between serum lipid levels and blood eosinophil counts in asthmatic individuals to date. The study population consisted of 2,544 National Health and Nutrition Examination Survey participants (2011–2018). In this investigation, HDL-C levels were adversely linked with blood eosinophil counts both before and after adjustment for sociodemographic, laboratory, personal life history, comorbidities, and medication use data, but serum LDL-C, total cholesterol or triglyceride was not correlated with blood eosinophil counts. LDL-C, age, BMI, triglyceride, and HDL-C were determined to be the five most important factors in blood eosinophil counts by machine learning of the XGBoost model. After controlling for confounders, the blood eosinophil counts fell by 45.68/uL for each increased unit of HDL-C (mmol/L), demonstrating a statistically significant difference. As a relatively essential variable, HDL-C was incorporated into generalized additive model and two-piecewise linear regression model to validate the robustness of the outcome.

Previous studies had shown the association between eosinophilia and dyslipidemia in general populations (12–14). A cross-sectional analysis included 417132 participants and demonstrated that a higher eosinophil count was associated with lower HDL cholesterol and apolipoproteinA-I (ApoA-I) concentration, as well as that higher triglyceride levels were associated with higher total leukocyte, basophil, eosinophil, monocyte, and neutrophil counts (12). Moreover, there are increasing studies finding an association between asthma and serum lipids (10, 15, 16). In 2015, Barochia et al. investigated the differences in lipid profiles between asthmatics and normal, healthy individuals. According to the findings of that study, atopic asthmatic patients had significantly higher HDL-C levels than nonatopic nonasthmatic patients (15). In 2017, Barochia and colleagues further indicated a positive association of triglycerides and a negative association of HDL-C with eosinophil count in atopic asthmatics (17), which was partially consistent with our results. However, their study solely focused on the connection and had a limited sample size. Our research indicates that HDL-C particles are inversely related to blood eosinophil counts in asthma. The durability of the results among asthmatics after correcting for many potential confounders, including the use of lipid-lowering medicines or antiallergic drugs, suggests that the relationship may be biologically significant (including antihistamines and glucocorticoids). In addition, we employed GAM to confirm the linear and negative association between HDL-C levels and blood eosinophil counts in asthmatic adults.

ACOS is a condition characterized by overlapping clinical features of both diseases. A meta-analysis of 19 studies indicated the prevalence of ACOS across studies to be even higher with an average of 26.5% (18). Previous studies had showed that airway and systemic eosinophilia are important treatable traits in both severe asthma and COPD (19). Luo et al. found that eosinophil levels were highly expressed in patients with asthma, patients with COPD and patients with ACOS (20). Moreover, Fricker’s study showed that molecular markers of type 2 airway inflammation do not differ between eosinophilic asthma and eosinophilic COPD (21). Therefore, we confer that blood eosinophil counts could also reflect the severity of ACOS which was consistent with asthma. There have been few reports regarding the relationship between serum lipid levels and blood eosinophil counts in ACOS. Gu et al. developed a model for predicting the severity of COPD. The study found that both eosinophil count and HDL were the factors that could distinguish between mild/moderate and severe (22). Our study was also the first study to explore the association between serum lipid levels and blood eosinophil counts in patients with ACOS. And we found the linear and negative relationship between HDL-C and blood eosinophil counts in adults with ACOS.

The findings that HDL-C particles are negatively associated with blood eosinophil counts in asthma, which are similar to the associations of HDL-C with lower cardiovascular risk (23), HDL not only mediates reverse cholesterol transport but also has anti-oxidant, anti-thrombotic, and anti-inflammatory functions (24). Endothelial lipase expressed in the lung could hydrolyze phospholipids, promote the catabolism of HDL particles, and regulate eosinophilic inflammation. In addition, the researchers found that endothelial lipase knockout mice had higher serum HDL levels after stimulation by allergens, and eosinophilic lung inflammation was alleviated (9). It has been proven that HDL and ApoA-I can inhibit antigen presentation-mediated T cell activation by disrupting lipid rafts in antigen-presenting cells (25). ApoA-I, the main apolipoprotein in HDL-C, has known functions including anti-inflammation and cholesterol efflux (26). Yao’s experiment discovered that apolipoprotein E or apolipoprotein A-I mimetic peptides could attenuate experimental asthma in the murine model, possibly by reducing pulmonary eosinophil infiltration (27, 28). These suggest that the anti-inflammatory properties of HDL and ApoA-I might be negatively correlated with blood eosinophil counts in asthmatics. And in our study, no significant differences were observed in relationships between LDL-C, total cholesterol, or triglyceride with blood eosinophil counts. This suggests that HDL-C might be related to the immune status of asthmatic adults.

Our study has several advantages over earlier research. First, our research provides a nationally representative, somewhat large sample of asthmatic adults with information on a number of potential confounding variables. Secondly, because confounders may influence the results, we use stratified analysis to identify the potentially protected group with higher HDL-C values. Then, we employ the machine learning of the XGBoost algorithm model to determine the relative importance of selected variables correlated with blood eosinophil counts. Using the generalized additive model, we observed the linear association between serum HDL levels and blood eosinophil counts. Although the precise relationship between HDL-C and eosinophil count remains unknown, the linkage has the potential to inspire the development of innovative asthma treatments. To elucidate the relationship between eosinophil count and serum lipid and to identify the underlying mechanism, additional research is required.

Nonetheless, we accept that our study has some limitations. Although the scope of our study is nationwide, the majority of the data is derived from the American population. As a result of unequal national development, dietary models may vary between countries. Due to the limitations of the cross-sectional study design, we cannot establish a causal association between serum lipid levels and blood eosinophil counts. Recent research has revealed, however, that free fatty acids directly impact myelopoiesis in the bone marrow *via* their release from bone marrow adipocytes (29), which then stimulates an increase in peripheral eosinophil counts. Next, we included asthmatic individuals based on questionnaire data rather than the lung function test. And the drugs included in our study that affected blood eosinophils were mainly cortisol drugs and other anti-allergy drugs, but no biologics. Therefore, additional prospective studies will be required in the future to shed light on the potential involvement of serum lipid in the control, progression, or treatment of asthma and to identify potential mechanisms of action.

## 5 Conclusion

This nationally representative study demonstrated that serum HDL-C was independently and inversely connected with blood eosinophil counts among American asthmatic adults, suggesting that serum HDL-C might be associated with the immunological status of asthmatic adults. However, there was no correlation between serum LDL-C, total cholesterol, or triglyceride levels and blood eosinophil counts. We hope that more individuals will recognize the significance lipids play in asthma, in the future.

## Data availability statement

Publicly available datasets were analyzed in this study. This data can be found here: http://www.cdc.gov/nchs/nhanes/.

## Ethics statement

The studies involving human participants were reviewed and approved by NCHS Research Ethics Review Board. The patients/participants provided their written informed consent to participate in this study.

## Author contributions

JW carried out the study design, data extraction and statistical analysis, drafted and revised the manuscript. MG conducted data extraction, statistical analysis, and revision of the paper. RZ carried out statistical analysis, drafted and revised the manuscript. CH conducted data extraction, drafted and revised the manuscript. SG participated in the study design, management of study and revision of the paper. All authors contributed to the article and approved the submitted version.
