# Supplementary material for: High density lipoprotein-cholesterol is inversely associated with blood eosinophil counts among asthmatic adults in the USA: NHANES 2011-2018
Source: Front Immunol. 2023 Apr 24;14:1166406. doi: 10.3389/fimmu.2023.1166406 (PMC10166227; doi:10.3389/fimmu.2023.1166406)
Supplement: Supplementary file 1 [file DataSheet_1.docx]

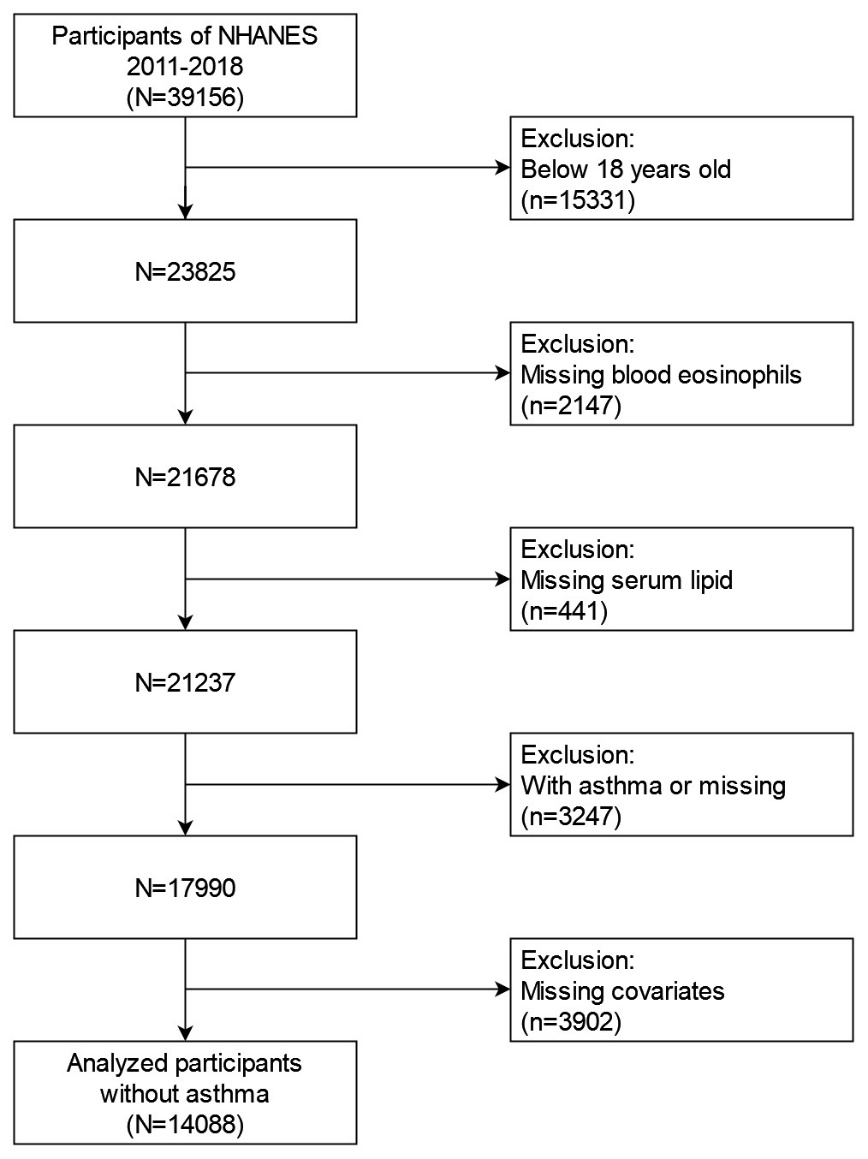


**Supplementary Figure 1.** Flowchart for choosing participants without asthma for analysis.


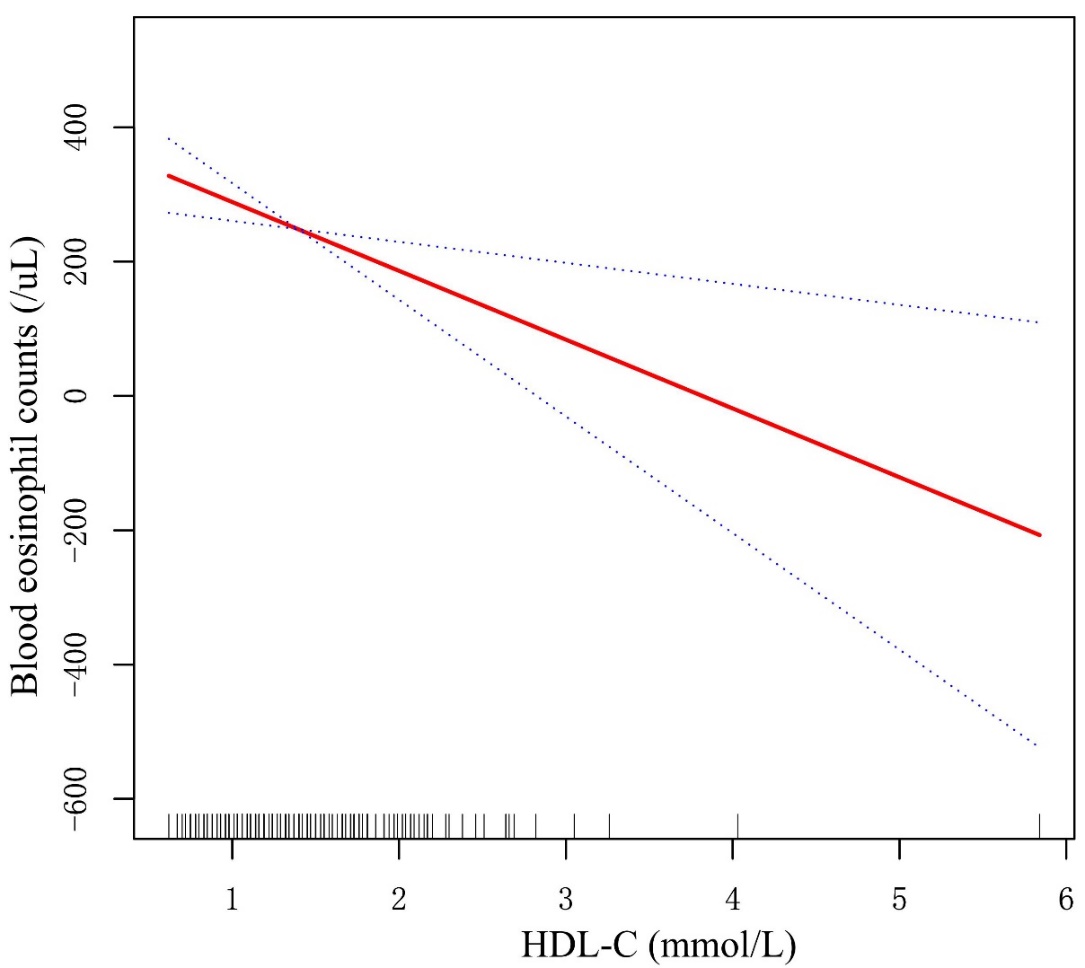


**Supplementary Figure 2.** Dose-response relationship of serum HDL-C levels with blood eosinophil counts among adults with Asthma-COPD Overlap Syndrome. The solid red line indicates the smooth fitting curve between serum HDL-C and blood eosinophil counts, whereas the dotted blue line represents 95% confidence intervals of the fitting.

| **Supplementary Table 1.** Weighted linear regression models and trend tests revealed the correlation between HDL-C and blood eosinophil counts among American adults without asthma. | | | |
| --- | --- | --- | --- |
|  | Model I | Model II | Model III |
|  | β (95% CI) P value | β (95% CI) P value | β (95% CI) P value |
| HDL-C | -53.85 (-63.03, -44.67) <0.0001 | -50.04 (-59.66, -40.42) <0.0001 | -32.45 (-42.01, -22.89) <0.0001 |
| HDL-C quartiles |  |  |  |
| Q1 | Reference | Reference | Reference |
| Q2 | -19.46 (-29.06, -9.87) 0.0002 | -17.54 (-27.36, -7.72) 0.0010 | -7.68 (-18.59, 3.23) 0.1760 |
| Q3 | -40.93 (-49.19, -32.68) <0.0001 | -36.89 (-45.63, -28.15) <0.0001 | -20.54 (-30.90, -10.18) 0.0004 |
| Q4 | -64.12 (-74.09, -54.15) <0.0001 | -59.83 (-70.46, -49.20) <0.0001 | -33.96 (-48.51, -19.42) 0.0001 |
| P for trend | <0.0001 | <0.0001 | <0.0001 |
| Note: Model I adjusted for none. Model II adjusted for age, race and gender. Model III adjusted for age, race, gender, education level, marital status, poverty to income ratio, BMI, smoked status, alcohol intake, lipid-lowering drugs, antiallergic drugs, hypertension, diabetes, COPD history, triglyceride, LDL-C and cholesterol.   \| **Supplementary Table 2.** Stratified correlation of serum HDL-C and blood eosinophil counts among adults with Asthma-COPD Overlap Syndrome. \| \| \| \| --- \| --- \| --- \| \| Subgroup \| N \| β (95% CI) P value \| \| **Gender** \|  \|  \| \| Male \| 120 \| -27.86 (-70.56, 14.84) 0.2093 \| \| Female \| 153 \| -52.08 (-74.55, -29.61) 0.0001 \| \| **Age** \|  \|  \| \| ＜40 \| 16 \| 220.83 (-407.85, 849.51) 0.5024 \| \| 40-60 \| 93 \| -60.10 (-125.98, 5.77) 0.0771 \| \| ＞=60 \| 164 \| -54.80 (-106.45, -3.15) 0.0391 \| \| **Race** \|  \|  \| \| Mexican American \| 14 \| 15.09 (-181.82, 212.01) 0.8831 \| \| Other Hispanic \| 17 \| 134.60 (-236.32, 505.52) 0.4878 \| \| Non-Hispanic White \| 172 \| -73.61 (-129.18, -18.04) 0.0103 \| \| Non-Hispanic Black \| 48 \| -52.09 (-123.05, 18.86) 0.1569 \| \| Other Race \| 22 \| 108.90 (-38.32, 256.11) 0.1626 \| \| **Education level** \|  \|  \| \| Less than high school \| 74 \| -63.95 (-167.88, 39.98) 0.2317 \| \| High school \| 64 \| -45.22 (-119.91, 29.47) 0.2399 \| \| More than high school \| 135 \| -53.77 (-106.19, -1.34) 0.0464 \| \| **Marital status** \|  \|  \| \| Married \| 119 \| -6.41 (-60.34, 47.51) 0.8161 \| \| Single \| 141 \| -124.38 (-186.75, -62.01) 0.0001 \| \| Living with a partner \| 13 \| 131.92 (-306.63, 570.46) 0.5674 \| \| **Poverty to income ratio** \|  \|  \| \| Low \| 91 \| -103.45 (-181.91, -24.98) 0.0114 \| \| Middle \| 90 \| -55.23 (-152.76, 42.31) 0.2701 \| \| High \| 92 \| -32.54 (-91.93, 26.85) 0.2858 \| \| **BMI** \|  \|  \| \| <25 \| 65 \| -67.58 (-162.86, 27.69) 0.1693 \| \| 25-28 \| 57 \| -59.67 (-134.30, 14.95) 0.1228 \| \| >=28 \| 151 \| -46.73 (-122.94, 29.47) 0.2313 \| \| **Lipid-lowering drugs** \|  \|  \| \| Yes \| 94 \| -46.70 (-136.20, 42.79) 0.3091 \| \| No \| 179 \| -56.44 (-104.04, -8.84) 0.0213 \| \| **Antiallergic drugs** \|  \|  \| \| Yes \| 110 \| -53.99 (-109.75, 1.77) 0.0604 \| \| No \| 163 \| -52.93 (-116.45, 10.59) 0.1044 \| \| Note: Due to the limited sample size after stratification, above stratified analyses adjusted for none. \| \| \| | | | |
